# The novel anticancer agent JNJ-26854165 is active in chronic myeloid leukemic cells with unmutated BCR/ABL and T315I mutant BCR/ABL through promoting proteosomal degradation of BCR/ABL proteins

**DOI:** 10.18632/oncotarget.13951

**Published:** 2016-12-15

**Authors:** Liangshun You, Hui Liu, Jian Huang, Wanzhuo Xie, Jueying Wei, Xiujin Ye, Wenbin Qian

**Affiliations:** ^1^ Institute of Hematology, The First Affiliated Hospital, College of Medicine, Zhejiang University, Hangzhou 310003, P.R. China; ^2^ Department of Hematology, The Fourth Affiliated Hospital, College of Medicine, Zhejiang University, Yiwu 322000, P.R. China

**Keywords:** BCR/ABL, T315I mutation, chronic myeloid leukemia, JNJ-26854165

## Abstract

Chronic myeloid leukemia (CML) is a clonal malignant disease caused by the expression of BCR/ABL. MDM2 (human homolog of the murine double minute-2) inhibitors such as Nutlin-3 have been shown to induce apoptosis in a p53-dependent manner in CML cells and sensitize cells to Imatinib. Here, we demonstrate that JNJ-26854165, an inhibitor of MDM2, inhibits proliferation and triggers cell death in a p53-independent manner in various BCR/ABL-expressing cells, which include primary leukemic cells from patients with CML blast crisis and cells expressing the Imatinib-resistant T315I BCR/ABL mutant. The response to JNJ-26854165 is associated with the downregulation of BCR/ABL dependently of proteosome activation. Moreover, in all tested CML cells, with the exception of T315I mutation cells, combining JNJ-26854165 and tyrosine kinase inhibitor (TKI) Imatinib or PD180970 leads to a synergistic effect. In conclusion, our results suggest that JNJ-26854165, used either alone or in combination with TKIs, represents a promising novel targeted approach to overcome TKI resistance and improve patient outcome in CML.

## INTRODUCTION

Chronic myeloid leukemia (CML) is a clonal malignant disease hallmarked by the expression of the BCR/ABL fusion protein that results from a reciprocal translocation involving chromosomes 9 and 22. BCR/ABL possesses a deregulated tyrosine kinase activity that drives a number of downstream signaling pathways, confers survival and proliferation advantages and restrains apoptosis, thus contributing to the pathogenesis of CML [1]. Imatinib mesylate, a small molecule tyrosine kinase inhibitor (TKI) that competitively binds to the ATP-binding site of BCR/ABL, is currently considered a first-line agent for the treatment of patients with newly diagnosed chronic-phase (CP) CML [2, 4]. Imatinib induces durable cytogenetic and molecular remission and prolonged lifespan in the majority of patients with CML [5], but approximately 20–30% of the patients eventually experience drug resistance, mainly as a consequence of mutations in the BCR/ABL kinase domain, genomic amplification of nonmutated BCR/ABL and BCR/ABL independence pathway [5–8]. The second-generation TKIs nilotinib, dasatinb, and bosutinib target most Imatinib-resistant BCR/ABL mutants. However, none of them is effective in patients with the T315I mutation [9]. In addition, all agents aimed at targeting the ATP-binding pocket of the BCR/ABL kinase domain alone do not eliminate CML stem cells [10–12].

The p53 tumor suppressor provides a potent barrier to cancerigenesis by inducing cell-cycle checkpoints, cellular senescence, or apoptosis in response to DNA damage and aberrant proliferative signals [13]. p53 mutations occur in approximately 50% of all cancers [14]. In human CML almost no p53 mutations is found, but in the blast-crisis phase (BC) and accelerated phase (AP) of CML more than 20% of patients display mutated p53 [15, 16]. Moreover, the function of wild-type (wt) p53 in CML can be disrupted by a sustained expression of MDM2, the crucial negative p53 regulator [17]. *In vitro* studies have shown that induction of p53 through MDM2 inhibition by the small-molecules such as Nutlins and MI219 effectively induces p53-mediated apoptosis in most blast crisis CML cells, with or without mutations including T315I variant [18, 19].

JNJ-26854165 (JNJ-165) is a novel small molecule that was initially thought to act as an antagonist to MDM2. [20, 21]. In a phase I trial performed in patients with refractory solid tumors, JNJ-165 displayed a modest anticancer activity and enabled p53 activation *in vivo* [22]. However, recent pre-clinical studies have demonstrated antiproliferative activity in various p53 wt and mutant cancer models [20, 23, 24], implying p53-independent activities. Thus, these two properties provide an advantage to prevent the selection of p53 mutant subclone in cancer during treatment of JNJ-165.

The aims of this study were to evaluate the efficacy of JNJ-165 in CML cells with or without p53 mutation *in vitro* and *in vivo* as a single agent and in combination with TKI and to confirm the mechanism of action of this potentially important drug in CML cells.

## RESULTS

### Antiproliferative and apoptotic effects of JNJ-165 in models of Imatinib-sensitive and-resistant CML

**Figure 1:**
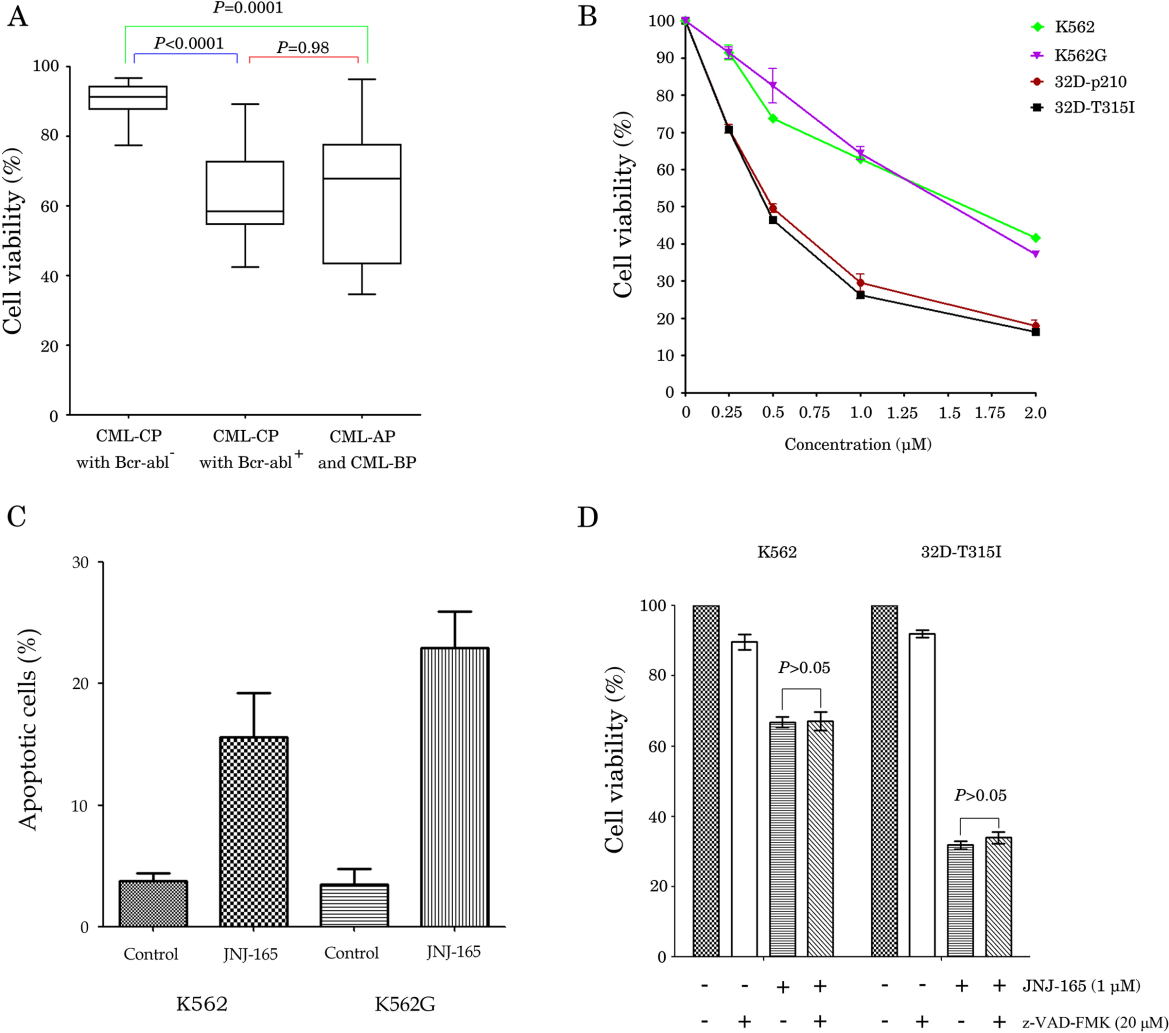
JNJ-165 inhibits proliferation and induces death in CML cell lines and primary cells (Imatinib-sensitive and -resistant) via caspase-independent pathway (**A**) The primary cells were obtained from CML patients, and were cultured with or without 2 μM JNJ-165 for 72 h, the viability of cells was assessed by an MTT assay. (**B**) CML cell lines K562, K562/G, 32D-BCR/ABL, and 32D-BCR/ABL-T315I, were cultured with or without different concentrations of JNJ-165 for 72 h. Growth inhibition by JNJ-165 was assessed by an MTT assay. Data were represented mean ± SD of three independent experiments. (**C**) CML Cell lines K562 and K562/G were harvested at 48 h after treatment with 2 μM JNJ-165. Cells were stained by an annexin V/PI-staining method and analyzed by flow cytometry. (**D**) Cell lines K562 and 32D-BCR/ABL-T315I were incubated for 6 h with 20 μM z-VAD-fmk, then exposed to 1 μM JNJ-165 for 72 h, the viability of cells was assessed by an MTT assay. Results are representative of three independent experiments and expressed as the mean ± SD.

We first examined the antiproliferation effect of JNJ-165 on primary cells from 24 newly diagnosed patients with CML, 9 patients with CML-AP/BC, and 13 cases with CML-CP treated with Imatinib or dasatinib, in whom expression of BCR/ABL mRNA determined by real time RT-PCR was very low or undetectable. The characteristics of the 46 CML patients analyzed in this study are detailed in Supplementary Table S1. CML primary cells were exposed to 2 μM JNJ-165 for 72 hours, the viability of cells from the CML-CP patients with BCR/ABL positive and CML AP/BP patients was reduced by 32.9% and 23.4%, respectively, compared with cells from the patients with very low or undetectable BCR/ABL (Figure 1A). We next evaluate the cytotoxicity of JNJ-165 to normal hematopoietic progenitor cells by colony formation assays. The results presented in Supplementary Figure S1 revealed that the number of hematopoietic colonies were not affected by JNJ-165. To investigate the effect of JNJ-165 on growth of CML cell lines, K562 and K562/G, an Imatinib-resistant cell line were incubated for 72 hours with escalating concentrations of JNJ-165. Cell viability of both cell types was inhibited with IC_50_ values of 1.54 and 1.67 μM, respectively (Figure 1B), suggesting similar sensitivity of these two cell lines to JNJ-165. Next, we treated a pair of murine 32D leukemic cell lines stably expressing wt or T315I mutant BCR/ABL (32D-BCR/ABL and 32D- BCR/ABL- T315I) with JNJ-165 and observed their growth remarkably inhibited, with IC_50_ values of 0.46 and 0.5 μM, respectively (Figure 1B). These data indicate that JNJ-165 is a potential agent to kill Imatinib-sensitive and resistant CML cells including cells with the T315I mutation.

To further clarify whether the antiproliferative activity of JNJ-165 was related to induction of apoptosis, Annexin V-FITC and PI double staining was performed. Treatment of K562 and K562/G cells with 2 μM JNJ-165 for 48 hours resulted in 15.6% and 22.9% apoptotic (annexin V^+^ and PI^+^) cells, respectively (Figure 1C). This is consistent with a previous report showing that JNJ-165 induced delayed apoptosis in p53 mutant cells including K562 cells [20]. However, JNJ-165 cytotoxicity against K562 and 32D-BCR/ABL-T315I cells was not significantly reduced by z-VAD-fmk pretreatment (Figure 1D), suggesting that cell death was caspase-independent.

### JNJ-165-induced cell death is p53-independent

**Figure 2:**
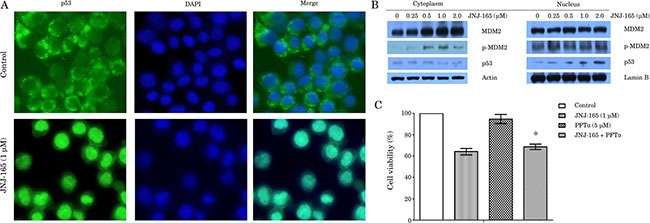
JNJ-165-induced CML cell lines death is p53-independent (**A**) Two-color fluorescence microscope analysis of antibody against p53 protein (green) and nuclei (DAPI staining, blue) showed nuclear accumulation of p53 in K562 cells with treatment of 1 μM JNJ-165, but not in control-treated cells after 48 h. Images were taken on a fluorescence microscopy (100×). (**B**) K562 cells was harvested at 48 h after treatment with various JNJ-165 concentrations, and then nuclear and cytoplasmic extracts were prepared to check the levels of p53, MDM2 and p-MDM2 by Western blotting. Actin and LaminB were used as loading control respectively. (**C**) Cell viability was assessed by MTT assay after pretreatment with 5 μM PFTα for 3 h followed by addition of 1 μM JNJ-165 in K562 cells. Combined treatment group did not display a significant reduction in cell death compared with JNJ-165 alone. Data are expressed as the mean ± SD of three independent experiments, and analyzed by ANOVA followed by the Tukey *t* test. *P > 0.05.

Since all CML cell lines used in this study had mutant-type p53 and JNJ-165 has been demonstrated to increase MDM2 and p53 levels in leukemia cells with wt p53 [20], we examined whether JNJ-165 affected this pathway in K562 cells. A small amount of p53 was diffusely distributed in the cytoplasm of untreated cells. After a 48-hour treatment with 2 μM JNJ-165, cells showed nuclear accumulation of p53 (Figure 2A). By examining intracellular distribution of MDM2 and p53 using Western blot analysis, we found that MDM2 protein and phospho-MDM2 (Ser166) were increased in the cytoplasm in a dose-dependent manner during JNJ-165 treatment, but not in the nucleus. In contrast, increased level of phospho-p53 was only observed in the nuclear fraction of cells treated with this agent (Figure 2B).

It was reported that pifithrin-α (PFTα), a chemical inhibitor of p53, reversibly blocks p53-transcriptional activity, preventing p53-mediated apoptosis [25]. In order to evaluate the possible role of p53 in the cytotoxicity of JNJ-165, we examined effect of PFTα on JNJ-165-induced cell growth inhibition. Pretreatment with PFTα for 3 hours followed by addition of JNJ-165 in K562 cells did not result in a significant reduction in cell death compared with JNJ-165 alone (Figure 2C). In view of these results, we further examined the inhibitory activity of Nutlin-3, a small-molecule antagonist of MDM2 that inhibits MDM2-mediated degradation of p53 [18, 26], against K562 and K562G cell lines. Although treatment with Nutlin-3 induced cell growth inhibition in these cells, IC_50_ values for K562 and K562G were 12.87 and 11.21 μM, respectively (Supplementary Figure S2). Taken together, these data suggest that killing of all tested CML cell lines with mutant p53 by JNJ-165 is bypass of p53 pathway.

### JNJ-165 depletes wt and mutant BCR/ABL and inactivates its downstream signaling

**Figure 3:**
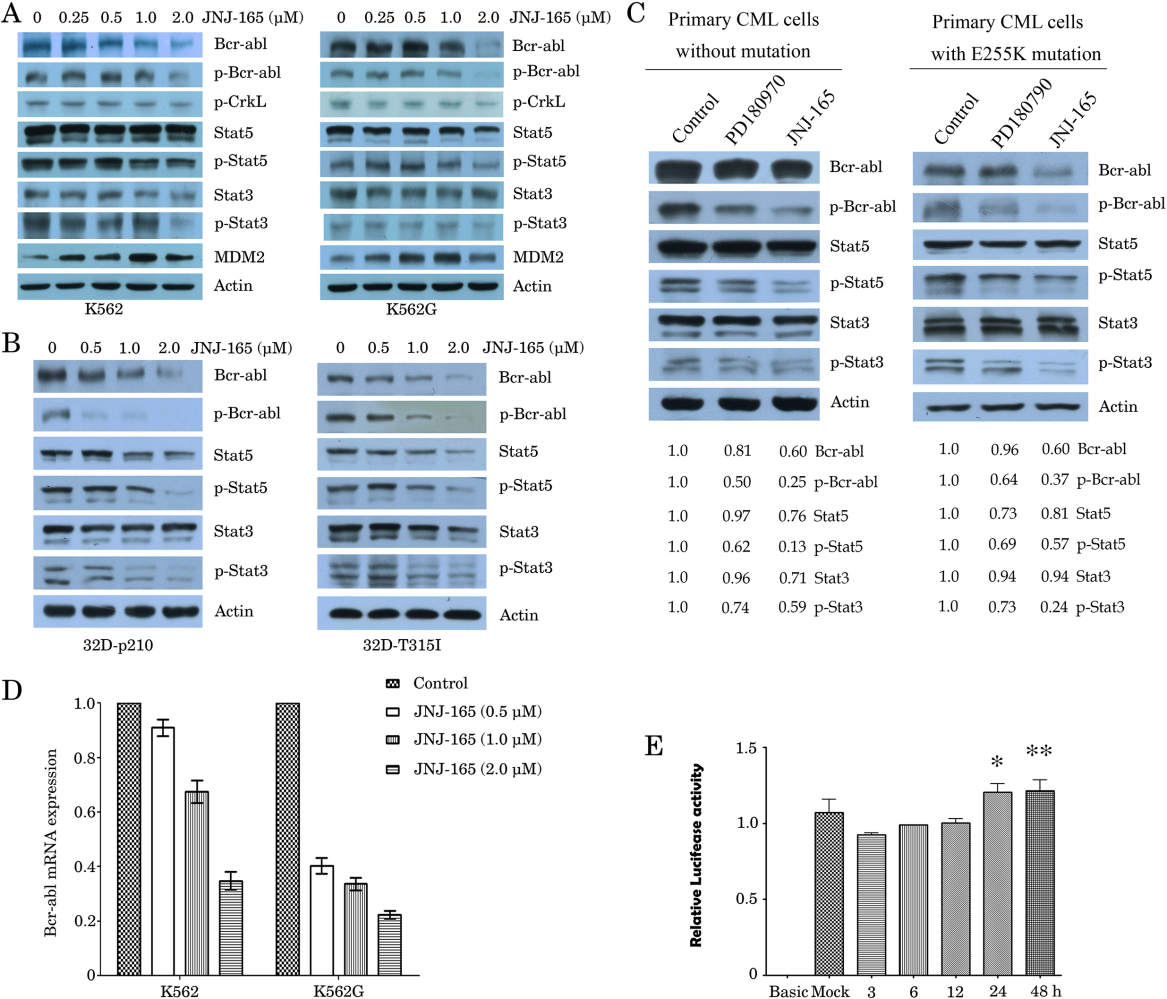
Investigations on the effect of JNJ-165 on promoter activity, mRNA level and protein expression of BCR-ABL (**A**) and (**B**) Four kinds of CML cell lines were treated with JNJ-165 at the indicated doses for 48 h. Whole-cell lysates were extracted to assess the levels of BCR-ABL and phosphorylated (p)-BCR-ABL (Tyr177), and then were analyzed for BCR-ABL downstream signaling mediators p-CrkL (Try207), total Stat5, p-Stat5 (Tyr694), total Stat3, and p-Stat3 (Ser727) expression by Western blot analysis. The expression of β-actin was used as loading control. The data are representative of three determinations with identical results. (**C**) The primary leukemic cells from a newly diagnosed CML patient without mutation and a primary CML patient harboring E255K mutation were treated with PD180970 (50 nM) or JNJ-165 (2 μM), and then protein expression was determined using Western blotting. JNJ-165 significantly inactivates p-BCR/ABL (wt/mutation) and its downstream signaling mediators. The difference in the levels of protein expression was semi-quantitatively determined by densitometry and expressed as a ratio. Actin was used as loading control. (**D**) RT-qPCR analysis monitoring BCR/ABL mRNA expression in K562 and K562G cells that were exposed to varying concentration of JNJ-165 for 48 h. Bars represent SD. of three independent experiments. (**E**) 293T cells were transfected with BCR promoter-luciferase report plasmid for 24 h, and then treated with 2 μM JNJ-165 for the indicated times. Cell lysates were analyzed for luciferase activity using the Luciferase Assay System Kit (Promega). Standard error was calculated from three independent experiments. *P > 0.05, 24 h vs control; **P > 0.05, 48 h vs control.

Next, we investigated the effect of JNJ-165 on the expression of BCR/ABL in CML cells by immunoblotting. Treatment with JNJ-165 for 48 hours resulted in not only decreased level of wt BCR/ABL in K562, K562G and 32D-BCR/ABL cells (Figure 3A and 3B) but also downregulation of T315I mutation BCR/ABL in 32D-BCR/ABL -T315I cells, in a dose-dependent manner (Figure 3B). To determine if the inhibition of total BCR/ABL protein leads to a decrease in its kinase activity, we evaluate the ability of JNJ-165 to inhibit the phosphorylation of BCR/ABL and its downstream targets CrkL and STAT3/5, which have been implicated in transformation and antiapoptotic signaling stemming from constitutive activation of the BCR/ABL kinase [27, 28]. Immunoblotting analyses showed that treatment of K562, K562G, 32D-BCR/ABL or 32D-BCR/ABL-T315I cells with JNJ-165 resulted in a potent decrease in the phosphorylated levels of BCR/ABL in accord with decrease in the total BCR/ABL protein (Figure 3A and 3B). Furthermore, at concentrations that correlated with its cytotoxic effects, JNJ-165 effectively inhibited the phosphorylation of STAT5 and STAT3. Phosphorylation of the BCR/ABL substrate CrkL was also downregulated, although to a lesser extent, in K562 and K562G cell lines (Figure 3A). Importantly, JNJ-165 (2 μM) was shown to significantly inhibit the phosphorylation of BCR/ABL and lower STAT5 and STAT3 kinase activity in both primary CML cells and primary CML cells harboring E255K, an Imatinib-resistant mutation [29, 30] (Figure 3C). Conversely to JNJ-165, PD180970, an inhibitor of protein tyrosine kinases against several imatinib-resistant mutations [31, 32], slightly decreased the amount of phosphorylated BCR/ABL and its downstream targets.

### JNJ-165 promotes the proteasomal degradation and reduces the mRNA of BCR/ABL

**Figure 4:**
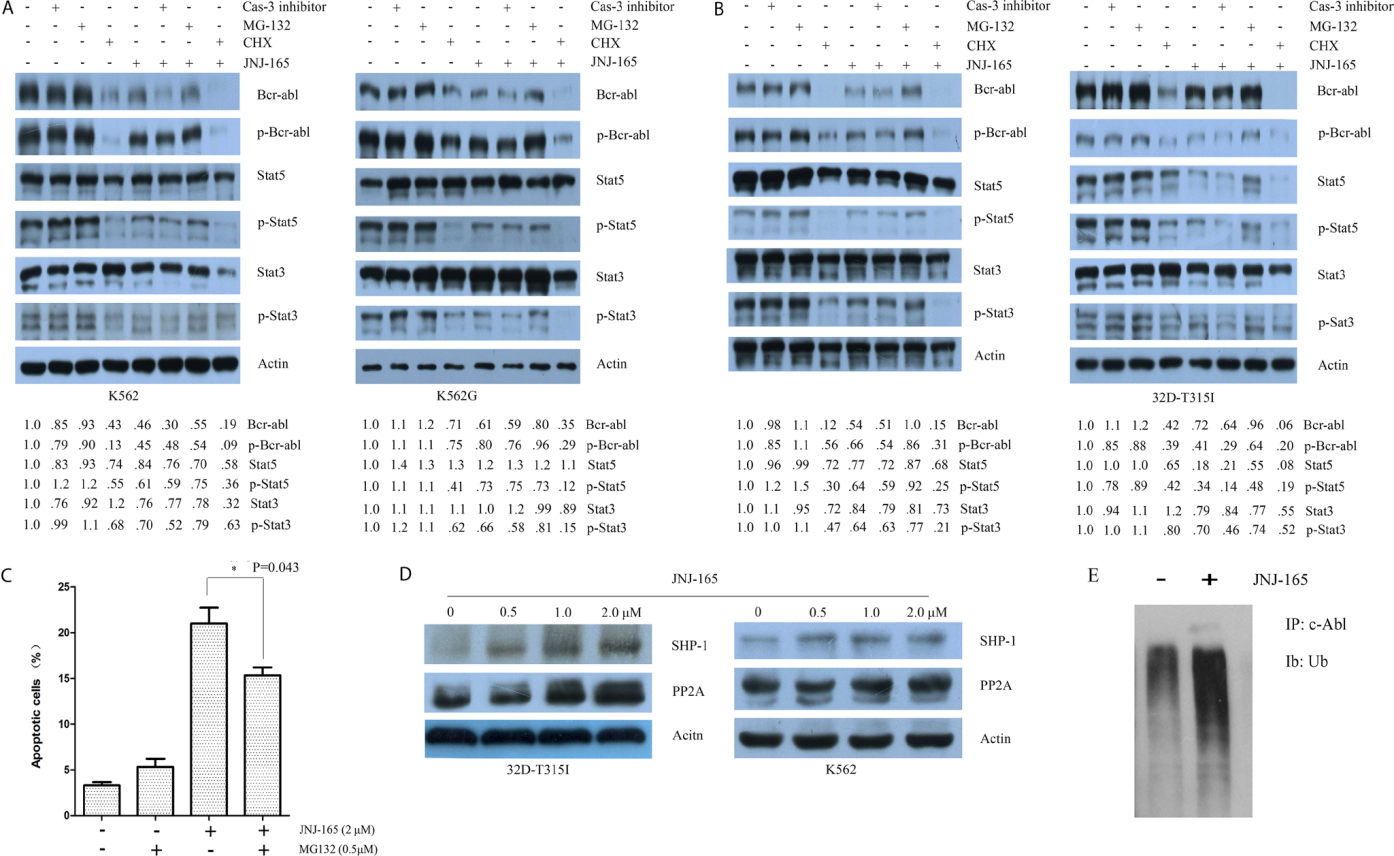
JNJ-165-mediated downregulation of BCR/ABL by promoting the proteosomal degradation pathway via PP2A-dependent mechanisms (**A**) and (**B**) Pretreatment with 2 h with z-DEVD-fmk (20 μM) or MG132 (0.5 μM ) or CHX (1 μg/ml), then exposed to 2 μM JNJ-165 for 48 h, whole cell proteins extracted from four kinds of CML cell lines were quantified and loaded. Western blotting was used to analyze the expression of total BCR/ABL, p-BCR-ABL (Tyr177), total Stat5, p-Stat5 (Tyr694), total Stat3, and p-Stat3 (Ser727). Actin was used as a loading control. The difference in the levels of protein expression was semi-quantitatively determined by densitometry and expressed as a ratio. (**C**) K562 cells were treated with JNJ-165 alone or combined with MG-132 (0.5 μM) for 48 h and apoptosis was determined by flow cytometry. (**D**) 32D-BCR/ABL-T315I and K562cells were treated with JNJ-165 at the indicated doses for 48 h and then analyzed for expression of SHP-1 and PP2A by Western blotting. Actin was used as a loading control. The results are representatives of three separate experiments. (**E**) Lysates from cells treated with PBS (control) or 2 μM JNJ-165 were subjected to immunorecipitations with anti-c-Abl. Immunoprecipitates were washed, resolved on gels, and immunoblotted for ubiquitin.

Attempts were then made to determine whether JNJ-165-mediated inhibition of BCR/ABL is because of repression of the BCR/ABL mRNA. K562 and K562G cells were exposed to varying concentration of JNJ-165 for 48 hours; real-time RT-qPCR analyses revealed that the mRNA transcripts of BCR/ABL were decreased in a dose-dependent fashion (Figure 3D). By using a luciferase reporter assay system, we studied the effect of JNJ-165 on the activity of the BCR promoter. Surprisingly, JNJ-165 did not induce significantly decreased transactivation of this promoter and that of AFP promoter (an unrelated gene control) (Figure 3E). Whether protein degradation through 26S proteasome contributes to JNJ-165-mediated decrease in the levels of BCR/ABL is not yet clear. To investigate this, we examined the effect of treatment with 2 μM JNJ-165 with or without the proteasome inhibitor MG-132 (0.5 μM) for 48 hours on the protein level of BCR/ABL in K562 and K562G cells. MG-132 could reverse the inhibition of BCR/ABL by JNJ-165 markedly (Figure 4A). Consistent with these results, apoptosis induced by JNJ-165 can be rescued partially by MG-132 (Figure 4C). As C-Abl is a substrate of caspase-3 [33], we checked the effect of caspase-3 inhibitor (z-DEVD-fmk) on JNJ-165-mediated downregulation of BCR/ABL, but found that z-DEVD-fmk did not restore the decline in BCR/ABL levels, excluding the possibility that JNJ-165-induced BCR/ABL decrease was dependent of caspase-3 activation. On the other hand, cotreatment of CML cells with JNJ-165 and the protein synthesis inhibitor cycloheximide (CHX) depleted the levels of BCR/ABL. Similar results were obtained with the 32D-BCR/ABL and 32D-BCR/ABL-T315I cells (Figure 4B).

The tumor suppressor phosphatase 2A (PP2A) is known to activate protein tyrosine phosphatase 1 (SHP-1), which catalyzes BCR/ABL dephosphorylation and proteosomal degradation [34, 35]. We, therefore, examined whether PP2A is regulated by JNJ-165 treatment. As shown in Figure 4D, JNJ-165 induced increased expression of PP2A in K562 and 32D-BCR/ABL-T315I cells in a dose-dependent fashion, which coincided with the upregulation of SHP-1. To determine whether the downregulation of Bcr-abl protein after JNJ-165 treatment was linked to its ubiquitination, protein lysates from JNJ-165-treated cells were immunoprecipitated with anti-c-Abl antibody and then followed by immunoblotting with anti-ubiquitin antibody. Results showed that JNJ-165 treatment of K562 cells led to a marked accumulation of polyubiquitinated c-Abl (Figure 4E). Collectively, these data indicate that JNJ-165 may trigger BCR/ABL degradation by the proteasome via PP2A-dependent mechanisms.

### Synergistic activity of JNJ-165 with TKIs Imatinib or PD180970

**Figure 5:**
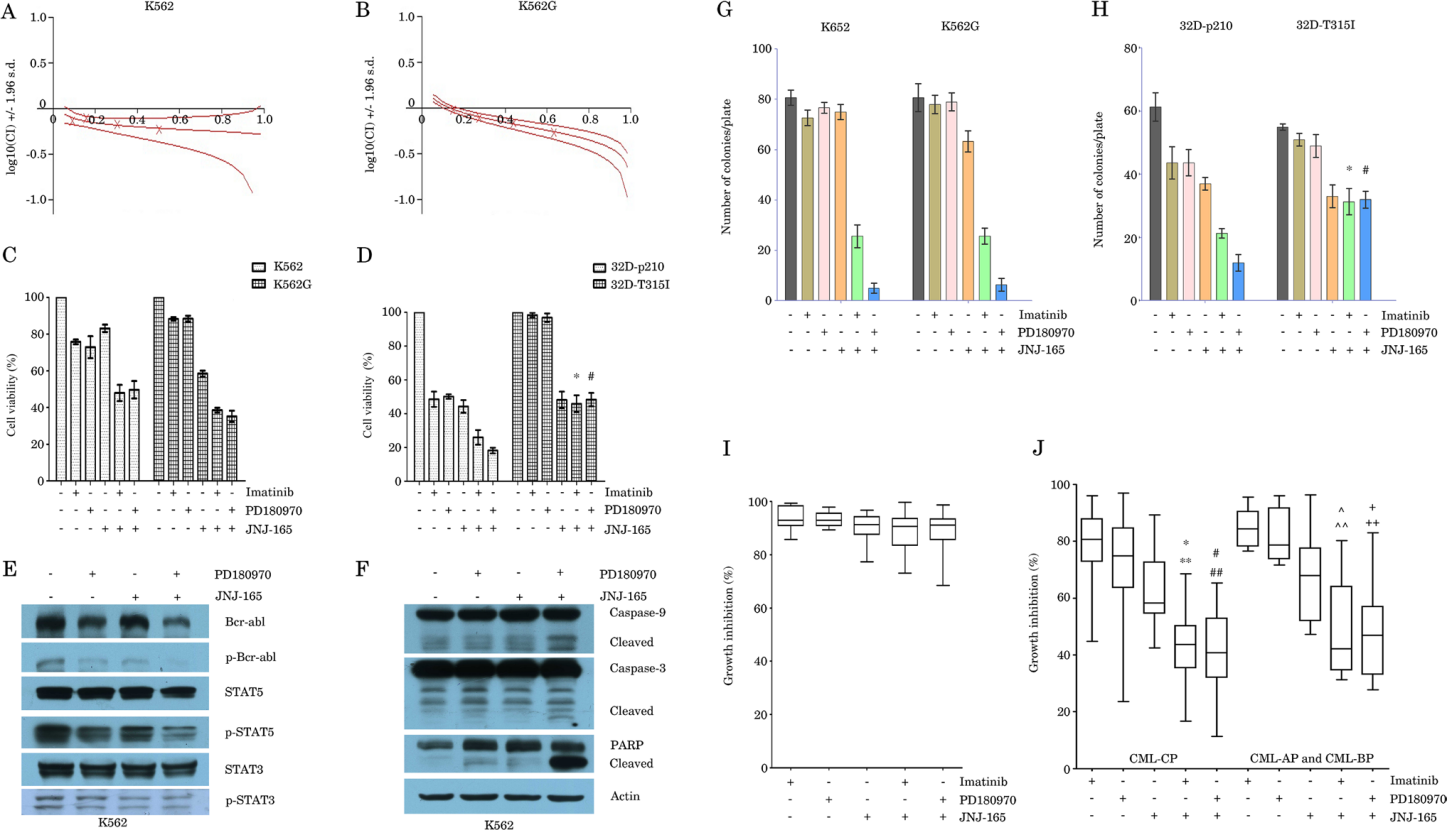
Enhanced suppression of CML cell lines and primary cells proliferation using the combination of JNJ-165 and Imatinib or PD180970 (**A**) and (**B**) K562 and K562/G cells were treated with a series of doses of JNJ-165 and PD180970 for 48 h. Growth inhibition was assessed by a MTT assay. The combination index (CIN) was calculated by Calcusyn software and expressed as log 10 (CIN). Where calculable, 95% confidence intervals are shown. The results were calculated from three independent experiments. (**C**) and (**D**) CML cell lines (Figure 5C for K562 and K562/G, 5D for 32D-BCR/ABL and 32D-BCR/ABL-T315I) were treated with JNJ-165 (2 μM), Imatinib (50 nM), PD180970 (50 nM), and JNJ-165-Imatinib, JNJ-165-PD180970 combination for 48 h. Cell viability was determined by a MTT assay. Mean ± SD. *n* = 3 independent experiments. *P > 0.05, ^#^*P* > 0.05. (**E**) and (**F**) K562 cells were treated with JNJ-165 (2 μM), PD180970 (50nM), and JNJ-165-PD180970 combination for 48 h. Whole-cell lysates were subjected to Western blotting analysis to examine the protein levels of total BCR/ABL, p-BCR-ABL (Tyr177), total Stat5, p-Stat5 (Tyr694), total Stat3, p-Stat3 (Ser727), caspase-9, caspase-3 and PARP. Actin was used as a loading control. The results are representatives of three independent experiments. (**G**) and (**H**) CML cell lines are treated with JNJ-165 (0.5 μM), Imatinib (12.5 nM) or PD180970 (12.5 nM) alone or in combination, and then plated in triplicate, at a density of 2000 cells/ml in methylcellulose medium. Colony counts at 7 days are shown as the average of three independent experiments; Bar ± SD (*n* = 3). *P > 0.05, ^#^*P* > 0.05. (**I**) and (**J**) Primary cells from patients with CML (Figure 5I for BCR/ABL negative or close to negative patients, Figure 5J for BCR/ABL positive patients) were treated with JNJ-165 (2 μM) alone or in combination with TKIs including Imatinib (50 nM) or PD180970 (50 nM) for 72 h, and then cell viability was measured by a MTT assay. *represents *P* < 0.0001/ *P* < 0.0001, JNJ-165-Imatinib vs JNJ-165/Imatinib; ^#^represents *P* < 0.0001/ *P* < 0.0001, JNJ-165—PD180970 vs JNJ-165/PD180970; ^represents *P* = 0.0407/ < 0.0001, JNJ-165-Imatinib vs JNJ-165/Imatinib; ^+^represents *P* < 0.0001/ *P* = 0.0243.

The combination of JNJ-165 and Imatinib or PD180970 was assessed by the MTT assay in K562, K562G, 32D-BCR/ABL, and 32D-BCR/ABL-T315I cell lines. First, K562 and Imatinib-resistant K562G cells were treated with a series of doses of JNJ-165 and PD180970 for 48 hours, and combination index (CI) was calculated by Chou and Talalay's method. A synergism was confirmed in both of these cell lines (Figure 5A and 5B). Similar result was also obtained with the cell lines treated with JNJ-165 in combination with Imatinib (Figure 5C). In 32D cells expressing wt BCR/ABL, all combinations of JNJ-165 with the TKIs significantly reduced cell proliferation compared with either agent alone (Figure 5D). However, in 32D cells harboring T315I mutation the cotreatment with JNJ-165 and either TKI did not result in a significant reduction in cell proliferation compared with JNJ-165 alone, while the imatinib or PD180970 alone treatment failed to inhibit cell growth (Figure 5D). This supports the view that CML cells containing T315I are resistant to both imatinib and PD180970 [6, 32].

The interactions between JNJ-165 and PD180970 were then examined in relation to perturbations in the BCR/ABL signaling pathway. Treatment with JNJ-165 or PD180970 alone modestly decreased the levels of BCR/ABL protein and phospho-BCR/ABL, but this effect was enhanced by coadminstration of JNJ-165 and PD180970. Furthermore, level of phospho-STAT5 was significantly decreased in cells exposed to both agents with respect to cells treated with JNJ-165 or PD180970 alone (Figure 5E). Consistent with the significant reduction in the levels of phospho-BCR/ABL and phospho-STAT5, an enhanced apoptosis, evidenced by activation of caspase pathway, was observed in K562 cells exposed to the JNJ-165 and PD180970 combination (Figure 5F).

Next, the impact of combined treatment of CML cell lines with JNJ-165 and Imatinib or PD180970 was determined in relation to effects on clonogenic survival (Figure 5G and 5H). Imatinib (12.5 nM) or PD180970 (12.5 nM) by itself had a slight effect on colony formation, whereas JNJ-165 (0.5 μM) administered alone reduced clonogenic survival by 6.25%, 23.5% and 39.3%, respectively in K562, K562G and 32D-BCR/ABL cell lines. However, all combinations of JNJ-165 with the TKIs resulted in a substantial reduction in clonogenicity (32.5% of control values for JNJ-165 plus Imatinib and 6.25% for JNJ-165 plus PD180970 in K562 cells). A similar synergistic effect was also observed in K562G and 32D-BCR/ABL cells, but not in 32D-BCR/ABL-T315I cells (*P* > 0.05).

Finally, we investigated if the combination of JNJ-165 and a TKI demonstrated a beneficial effect in primary cells from patients with CML. Mononuclear cells derived from bone marrow of patients with CML-CP and CML-AP or BP and from healthy individuals were treated with JNJ-165, Imatinib or PD180970 alone, or with JNJ-165 in combination either TKI for 72 hours, and cell viability was measured. Consistent with data from the cell lines, JNJ-165 and either TKI synergized to inhibit the proliferation of cells expressing BCR/ABL from newly diagnosed patients with CML-CP and from patients with CML-AP or BP (Figure 5J). However, the primary cells from CML patients treated with Imatinib or dasatinib, in whom the level of BCR/ABL gene expression was very low or negative, were resistant to JNJ-165, the TKIs, and combination therapy with JNJ-165 and either TKI (Figure 5I). As a control, we observed the number of colony forming units (CFUs) after treatment with JNJ-165, PD180970 or their combination in the primary cells from healthy individuals (*n* = 4) were also resistant to the above treatment (data not shown).

### JNJ-165 and TKIs induce synergetic lethality in CML cells, but not in T315I cells *in vivo*

**Figure 6:**
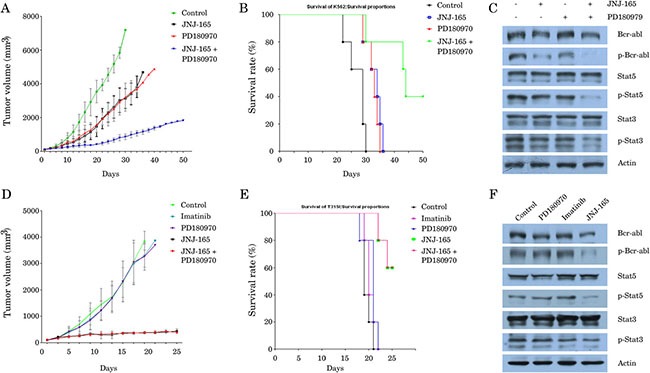
The synergetic lethality of JNJ-165 combined with TKIs in CML cells, but not in T315I cells in vivo SCID mice bearing subcutaneous K562 xenografts (*n* = 10) were randomized to receive one of the following treatments on 7 consecutive days: PBS, JNJ-165 (40 mg/kg/d), PD180970 (50 mg/kg/d), or JNJ-165 combined with PD180970. Whereas, the animals with 32D-BCR/ABL-T315I xenograft (*n* = 10) were divided into five groups and treated as follows: PBS, JNJ-165 (40 mg/kg/d), Imatinib (50 mg/kg/d), PD180970 (50 mg/kg/d), or JNJ-165 combined with PD180970. Tumor size and volume in xenografts were measured and are presented as the mean ± SD (Figure (**6A**) for K562, (**6D**) for 32D-BCR/ABL-T315I). Kaplan-Meier survival curves for mice with K562 xenografts treated with different drugs (Figure (**6B**) for K562, (**6E**) for 32D-BCR/ABL-T315I). Tumor tissues were obtained on day 5 after treatment, and the levels of BCR/ABL protein, phospho-BCR/ABL and downstream molecules were determined by Western blotting. The expression of actin was used as loading control (Figure (**6C**) for K562, (**6F**) for 32D-BCR/ABL-T315I).

We assessed the implications of our findings *in vivo* by using two different animal models: K562 and 32D-BCR/ABL-T315I xenografts in SCID mice. The growth of tumors established from K562 cells was significantly suppressed in the mice treated with either JNJ-165 or PD180970 as compared with the control mice (*P* < 0.0001; Figure 6A). However, combination treatment showed an impressive antitumor activity. The mean tumor volumes of animals treated with JNJ-165 or PD180970 were 3033.2 mm^3^ and 2932.1 mm^3^, respectively on day 27 after start of therapy. In marked contrast, the mean tumor volume of animals cotreated with two drugs was 805.4 mm^3^. Consistent with these results, the combination therapy dramatically prolonged survival compared with each agent alone (Figure 6B; *P* = 0.0269). Furthermore, the levels of BCR/ABL protein, phospho-BCR/ABL and downstream molecules were significantly lower in co-treated tumor than in tumors treated with JNJ-165 or PD180970 (Figure 6C), which may contribute to combination therapy cytotoxicity.

It is important to note that neither PD180970 nor Imatinib were growth inhibitory to the 32D-BCR/ABL-T315I tumors (Figure 6D) and prolonged the survival of mice (Figure 6E). In contrast, JNJ-165 alone caused an almost complete regression of tumors established from 32D-BCR/ABL-T315I cells and significantly prolonged the survival compared with untreated control (*P* = 0.0015). All control mice died within 21 days, while all mice treated with PD180970 or Imatinib died before day 22. Whereas, only two out of five mice (40%) receiving JNJ-165 died within 25 days because of gastrointestinal toxicity such as asthenia and anorexia (Figure 6E). As expected, significant downregulation of BCR/ABL and downstream molecules was observed in the section from JNJ-165-treated animal, but not in tumors from mice treated with PD180970 or Imatinib (Figure 6F). Additionally, there were no synergetic effects of JNJ-165 and PD180970 on tumor growth and animal survival. Taken together, these data indicated that a critical role of JNJ-165 is required in order to achieve a therapeutic effect.

## DISCUSSION

Although TKIs has revolutionized drug therapy of CML, the development of drug resistance and persistence of TKI-insensitive leukemic stem cells remain obstacles to eradicating the disease. The MDM2 has become a validated target for cancer treatment because of its important role in negative regulation of p53 [36]. A number of recent studies have reported that small molecular inhibitors of MDM2 induce apoptosis in BCR/ABL expressing cells, including CML blast crisis cells regardless of the presence of the T315I mutation [18, 19]. JNJ-165 is a novel tryptamine derivative with p53-activating properties reported act as a MDM2 inhibitor with *in vitro* or *in vivo* efficacy in acute leukemia, lymphoma, and solid tumors [20, 22, 24]. In this study, we have evaluated JNJ-165 in a variety of CML cell lines with p53 mutation, including those resistant to Imatinib and harboring T315I mutation, and primary CML cells, most of them being wild type p53 [15, 16]. We found that, unlike the selective MDM2 antagonist Nutlin-3 that was not active in K562 and K562G cells, JNJ-165 showed a strong activity against all tested cell lines containing mutation p53 and primary CML cells. JNJ-165 also significantly inhibited the growth of Imatinib-resistant BCR/ABL-T315I as well as Imatinib-sensitive CML cells in mouse xenografts. Although some p53 induction was observed in K562 cells treated with JNJ-165, PFTα, an inhibitor of p53, did not prevent JNJ-165-mediated cell death, suggesting that cell death induced by JNJ-165 is independent of the MDM2-p53 interaction in human CML cells. The most notable effect on cells was an inhibition of BCR/ABL in both cells bearing wild-type BCR/ABL and T315I mutant BCR/ABL. Taken together, these data suggest that JNJ-165 could be a useful therapeutic agent in CML.

Recently, some studies have suggested that promoting the degradation of BCR/ABL represents an alternative approach to treat CML. For example, arsenic was shown to induce ubiquitination and degradation of BCR/ABL via upregualtion of c-CBL, a RING-type E3 ligase [37]. In addition, the small molecular deubiquitinase inhibitor WP1130 and histone deacetylase inhibitor LAQ824 were also found to induce BCR/ABL ubiquitination, which results in a reduction in BCR/ABL signaling and promotes CML cell apoptosis [38, 39]. In this study, we observed that JNJ-165 decreased mRNA of BCR/ABL. However, our data did not support that JNJ-165 is a transcription inhibitor, because JNJ-165 treatment fail to affect the BCR/ABL promoter. Additionally, JNJ-165-mediated down-regulation of BCR/ABL was independent of caspase activation. Based on these, we investigated whether BCR/ABL protein degradation was pathways for JNJ-165 actions, and found that cotreatment with MG-132 reversed the JNJ-165-mediated inhibition of the BCR/ABL protein. Furthermore, we demonstrated that JNJ-165 treatment led to a marked accumulation of polyubiquitinated c-Abl. Western blotting analysis showed a significant increase of PP2A and SHP-1 protein levels in cells treated with JNJ-165, as compared with the untreated control. The serine/threonine phosphatase 2A (PP2A) functions as a tumor suppression by activating protein tyrosine phosphates 1 (SHP-1), which catalyzes BCR/ABL dephosphorylation and proteosomal degradation [35]. However, the PP2A protein is usually inactivated in CML cells because BCR/ABL stimulates prevention of its auto-dephosphorylation at tyrosine 307 [35, 40] and cancerous inhibitor of PP2A (CIP2A) that has been shown to be overexpressed in CML patients destined to progress to blast crisis mediates inhibition of PP2A [41]. Neviani *et al*., [42] demonstrated that PP2A-activating agents such as FTY720 selectively reduced survival and self-renewal of TKI-resistant CML stem cells, through BCR/ABL kinase-independent pathway. Taken together, our data suggest that activation of SHP-1-PP2A pathway may contribute to the cytotoxic action of JNJ-165. However, further studies are required to elucidate the exact mechanism by JNJ-165 deregulate SHP-1-PP2A pathway.

Combination therapy is necessary for cancer because tumors are genetically diverse and drug-resistant seems inevitable [43]. Having established an effect for JNJ-165 as a single agent in p53 mutant CML cells, we also investigated the possibility of combining JNJ-165 with the TKIs Imatinib or PD180970 that is active against several BCR/ABL mutations [31, 32], so as to reinforce their respective antileukemic activities. We showed that JNJ-165 significantly and synergistically increased TKIs oncotoxicity both in CML cell lines with p53 mutant and primary CML cells with BCR/ABL-positive, while the combination was not significant toxic for MNCs from BCR/ABL-negative CML patient. Notably, this combination markedly enhanced the inhibition of BCR/ABL expression and phosphorylation, indicating a critical role of the BCR/ABL signaling in mediating the synergy of JNJ-165 plus TKI. However, in CML cells harboring BCR/ABL-T315I mutation, all combinations of JNJ-165with the TKIs did not increase cell death compared with JNJ-165 alone. These results support the notion that CML cells with T315I mutation are resistant to most of the TKIs, except for ponatinib [44, 45]. Based on these observations, we concluded that the combined treatment with JNJ-165 and a TKI might offer a novel therapeutic strategy not only for p53 wild-type CML patients, but also for the patients with p53 mutation that is detected in about 20% of patients in blast crisis [15, 16].

In conclusion, we have shown that MDM2 inhibitor JNJ-165 induces cell death in a variety of CML cell lines with p53 mutation, including those resistant to Imatinib and harboring T315I mutation. Furthermore, we have identified a novel mechanism of action of JNJ-165. Our results suggest that JNJ-165, used either alone or in combination with TKIs, represents a promising novel targeted approach to overcome TKI resistance and improve patient outcome in CML.

## MATERIALS AND METHODS

### Reagents and antibodies

Imatinib mesylate (STI571), Nutlin-3, MG-132, PFTα, and CHX were purchased from Sigma (St Louis, MO, USA), and JNJ-165 and PD180970 were purchased from Selleckchem (Houston, TX, USA). The caspase general inhibitor z-VAD-fmk and casepase-3 inhibitor z-DEVD-fmk were obtained from Biovision (Mountain View, CA, USA). All primary antibodies used in this study were purchased from Cell signaling Technology (Beverly, MA, USA), except for the human anti-p53 (ab32380; Epitomics, Burlingame, CA, USA), anti-phospho-p53 (Epitomics), lamin B (Proteintech Group, Chicago, IL, USA), anti-Abl and actin (Santa Cruz, Santa Cruz, CA, USA).

### Patient samples and cell lines

Heparinized bone marrow samples from the patients with CML were obtained after informed consent and with the approval of the Ethics Committee of Zhejiang University the First Affiliated Hospital (Hangzhou, China). The main clinical data of the patients were abstracted from clinical records (Supplementary Table 1). Bone marrow mononuclear cells (MNCs) were isolated by gradient centrifugation with lymphocyte cell separation medium. Human CML cell line K562 was obtained from American Type Culture Collection (ATCC, Rockville, USA). K562/G, an Imatinib-resistant cell line, was kindly provided by Institute of Hematology, Chinese Academy of Medical Sciences (Tianjin, China) [46]. 32D-BCR/ABL, an Imatinib-sensitive murine CML cell line carrying a WT ABL gene, and 32D-BCR/ABL-T315I, an Imatinib-resistant CML cell line carrying a T315I mutation in BCR/ABL were provided by Prof. L Qiu, (Harbin Institute of Hematology & Oncology, Harbin, China) [47]. DNA was extracted from all cell lines tested for genomic sequence analysis of p53 mutation [48]. Cells were cultured in RPMI 1640 with 10% fetal bovine serum (FBS, Hyclone, Utah, USA) at 37°C and 5% CO_2_ as described [49].

### Cell viability

For cell proliferation assays, leukemia cells were seeded into 96-well plates at a density of 15 × 10^4^ cells/ml and then treated with different agents at the indicated doses in the figure legends. After a 48-hour or 72-hour incubation period, 20 μl of 3-(4,5-dimethylthiazol-2-yl) -2,5-diphenyltetrazolium bromide (MTT; Sigma) solution (5 mg/ml) was added into each well and cells were cultured for an additional 4 hours at 37°C, then the supernatants were removed and 200 μl DMSO (Sigma) was used to dissolve the formazan crystals. Absorbance was read at 570 nm with a microplate reader (Bio-RAD, Berkeley, USA). Each assay was performed three times in triplicate.

### Leukemic colony-forming cell assay

AML cells were cultured in 0.8% methylcellulose as described previously [49]. Briefly, 2 × 10^3^ cells were placed in 35-mm tissue culture dishes containing Iscove's modified Dulbecco's (IMDM) medium supplemented with 20% FBS (Hyclone), 100 μM ß-mercaptoethanol, and methylcellulose at final concentration of 0.8%. After 10 to 12 days incubation at 37°C the number of colonies containing more than 20 cells was counted under an inverted microscope (Olympus).

### Assessment of apoptosis

Detection of apoptosis was performed by mean of the annexin V-FITC and propidium iodide (PI) detection kit (BD Pharmingen, San Diego, CA, USA). Briefly, K562 and K562G cells were treated with JNJ-165 (2 μM) for 48 hours, washed with PBS buffer containing 5 mmol/L EDTA, incubated in the dark at 4°C with annexin V-FITC and PI for 30 min, and then analyzed with FACScan flow cytometer and CELL Quest software (Becton Dickinson, Franklin Lakes, USA).

### Western blot analysis

After treatment, cytoplasmic and nuclear extracts were prepared using Nuclear/Cytosol Fractionation Kit (BioVision) according to the manufacturer's manual, and fifty μg samples of nuclear, cytoplasmic, or whole cell extracts were subjected to Western blotting as previously described [49].

### Immunoprecipitation of Bcr-Abl and immunoblot analysis

Following the treatment with JNJ-165, cells were lysed in lysis buffer for 30 min on ice, and the nuclear and cellular debris were cleared by centrifugation. Cell lysates (100 μg) were incubated with Abl-specific monoclonal antibody for 1 h at 4°C. To this mixture, washed protein G-agarose beads were added and incubated overnight at 4°C. The immunoprecipitates were washed three times in the lysis buffer, and proteins were eluted with the SDS sample loading buffer prior to immunoblot analyses with specific antibodies against anti-ubiquitin antibody (Santa Cruz).

### BCR promoter luciferase reporter assay

Luciferase report vector of BCR promoter was constructed using pGL-2-basic vector as following: BCR promoter fragment (−816 to +71) was amplified by PCR using genomic DNA extracted from K562 cells, which contain the Philadelphia chromosome, with forward primer 5′-ATCTCGAGCTTGGGGACACGCGGCTGGA-3′ and reverse primer 5′-ATAAGCTTCATGCGCGGGGCTCT GAGT-3′, designed to include an Xho I site and a Hind III site, respectively. The PCR product was cloned into pGL2-basic vector (Promega, Madison, WI, USA), and the DNA sequence was confirmed using a 3730 DNA Analyzer (ABI, Applied Biosystems, Foster City, CA, USA). To test whether JNJ-165 affect the activity of BCR promoter, 293T cells were transfected with BCR promoter-luciferase report plasmid for 24 hours, and then treated with 2 μM JNJ-165 for the indicated times. After treatment with JNJ-165, cell lysates were analyzed for luciferase activity using the Luciferase Assay System Kit (Promega).

### Reverse transcription quantitative real-time PCR (RT-qPCR)

Total cellular RNA was purified using the RNeasy Mini Kit (Qiagen, Valencia, CA, USA). Reverse transcription was performed with random hexamers using Superscript first-strand synthesis system (Invitrogen, Carlsbad, CA, USA) according to the manufacturer's instructions. All PCR reactions were carried out on the iQ5 thermal cycling system (Bio-Rad Laboratories, Hercules, CA, USA). Optimal reaction conditions for amplification of both BCR/ABL andβ-actin were as follows: 30 sec at 95°C for initial denaturing, 10 sec at 95°C for denaturing and 60 sec at 60°C for annealing and extension for a total of 40 cycles. The PCR primers were as follows: BCR/ABL-sense: 5′-TCCGCTGACCATCAAYAAGGA-3′; antisense: 5′-CACTCAGACCCTGAGGCTCAA-3′; β-actin -sense: 5′-GTCATCACCATTGGCAATGAG-3′; antisense: 5′- CGTCACACTTCATGATGGAGTT -3′. Fluorescence data from each sample were analyzed with the 2^−ΔΔCt^ method as described in detail elsewhere [49].

### Immunofluorescence assay

The p53 protein localization was examined by Immunofluorescence assay. K562 cells were treated with PBS or with JNJ-165 (1 μM) for 48 hours, washed in PBS, then fixed in 4% paraformaldehyde, blocked with PBS containing 2% goat serum, and 0.2% Triton X-100. After removing the blocking buffer, cells were incubated with anti- human p53 primary antibody overnight. Primary antibody was detected by Texas Green-conjugated goat anti-rabbit antibody (Santa Cruz). Cells were counterstained with DAPI (Southern Biotech, Birmingham, AL, USA).

### *In vivo* tumor xenograft study

Sever combined immunodeficient (SCID) mice were obtained from the Shanghai Experimental Animal center of the Chinese Academy of Sciences (Shanghai, China). Human K562 cells or 32D-BCR/ABL-T315I cells were subcutaneously injected into right flank of each mouse, respectively. When tumor's volume reached 100–120 mm^3^, the animals were randomized into treatment groups. Tumor growth was monitored and measured as described previously [50]. For K562 xenografts, the mice were divided into four groups and treated as follows: PBS, JNJ-165 (40 mg/kg/d), PD180970 (50 mg/kg/d), or JNJ-165 combined with PD180970. Whereas, the animals with 32D-BCR/ABL-T315I xenograft were divided into five groups and treated as follows: PBS, JNJ-165 (40 mg/kg/d), Imatinib (50 mg/kg/d), PD180970 (50 mg/kg/d), or JNJ-165 combined with PD180970. The agents used in this study were administered by gavage for seven consecutive daily, and dosages were obtained from previously reported studies [24, 50]. Tumor specimens from one of each groups were harvested at five days after treatment, and then were analyzed by Western blotting for the expression of BCR/ABL, STAT3 and STAT5.

### Statistical analysis

All experiments were performed in triplicates, and the results were presented as means ± SD. Data were statistically evaluated by ANOVA and Tukey's test using Prism version 2.0 software (GraphPad Software, San Diego, CA, USA). Synergisms in the combination treatments were analyzed using a commercially available software program (CalcuSyn, Biosoft, Cambridge, UK). *A P*-value of < 0.05 was considered significant.

## SUPPLEMENTARY MATERIALS FIGURES AND TABLE


