# Supplementary material for: The novel anticancer agent JNJ-26854165 is active in chronic myeloid leukemic cells with unmutated BCR/ABL and T315I mutant BCR/ABL through promoting proteosomal degradation of BCR/ABL proteins
Source: Oncotarget. 2016 Dec 15;8(5):7777–90. doi: 10.18632/oncotarget.13951 (PMC5352360; doi:10.18632/oncotarget.13951)
Supplement: Supplementary file 1 [file oncotarget-08-7777-s001.pdf]

The novel anticancer agent JNJ-26854165 is active in chronic myeloid leukemic cells with unmutated BCR/ABL and T315I mutant BCR/ABL through promoting proteosomal degradation of BCR/ABL proteins

Supplementary Materials

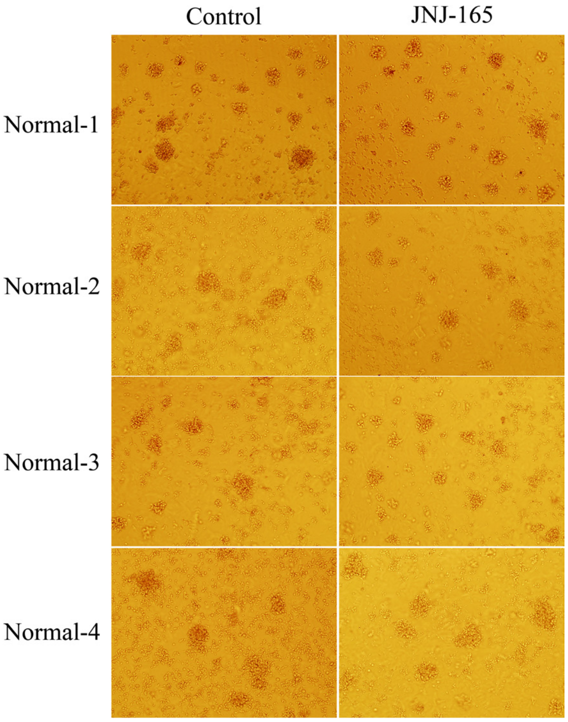

Supplementary Figure S1: The cytotoxicity of JNJ-165 on normal hematopoietic progenitor cells. Mononuclear cells obtained from bone marrow of four healthy volunteers were incubated in methylcellulose medium supplemented with recombinant human granulocyte-macrophage colony-stimulating factor, stem cell factor and interleukin 6 for seven days, at which time colonies were visualized with an microscope.

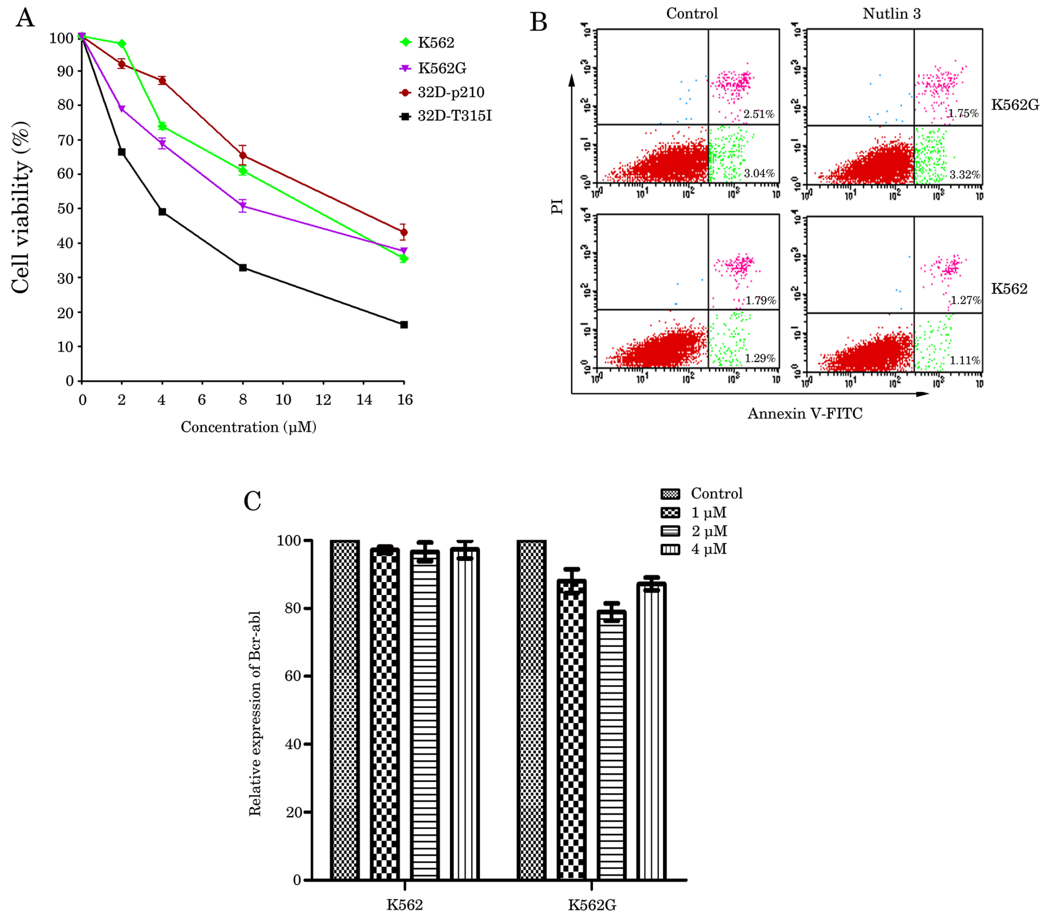

Supplementary Figure S2: The inhibitory activity of Nutlin-3 against CML cell lines. (**A**) Four kinds of CML cell lines were treated with Nutlin-3 at the indicated doses for 72 h. Growth inhibition by Nutlin-3 was assessed by an MTT assay. Data were represented mean ± SD of three independent experiments. (**B**) CML Cell lines K562 and K562/G were harvested at 48 h after treatment with 5 μM Nutlin-3. Cells were stained by an annexin V/PI-staining method and analyzed by flow cytometry. (**C**) RT-qPCR analysis monitoring BCR/ABL mRNA expression in K562 and K562G cells that were exposed to varying concentration of Nutlin-3 for 48 h. Bars represent SD. of three independent experiments.

Supplementary Table S1: Clinical characteristics of CML patients

Patients	Sex/age, y	Diagnosis	Prior treatment	Bcr/abl expression	Mutation
1	M/25	CML-CP	Imatinib	negative	
2	F/70	CML-CP	Imatinib	negative	
3	F/43	CML-CP	Imatinib	negative	
4	F/31	CML-CP	Imatinib	negative	
5	M/34	CML-CP	Imatinib	negative	
6	M/48	CML-CP	Imatinib	negative	
7	F/33	CML-CP	Imatinib	negative	
8	M/24	CML-CP	Imatinib	negative	
9	F/52	CML-CP	Imatinib	0.35%	
10	M/58	CML-CP	Imatinib	negative	
11	F/29	CML-CP	Imatinib	negative	
12	M/43	CML-CP	Imatinib	negative	
13	M/62	CML-CP	Imatinib	negative	
14	F/72	CML-BC	Imatinib Dasatinib	82%	Y253H,E255K
15	F/58	CML-BC	Imatinib	87%	M244V
16	F/57	CML-AP	Hydroxyurea	76%	negative
17	M/32	CML-AP	Hydroxyurea	43%	negative
18	F/44	CML-AP	Hydroxyurea	114%	negative
19	M/48	CML-BC	Imatinib	90%	G250E
20	M/39	CML-AP	Hydroxyurea	120%	negative
21	M/65	CML-AP	Hydroxyurea	47%	negative
22	M/32	CML-BC	Imatinib	150%	negative
23	M/47	CML-CP	Newly diagnosed	130%	
24	M/41	CML-CP	Newly diagnosed	107%	
25	M/47	CML-CP	Newly diagnosed	61%	
26	M/22	CML-CP	Newly diagnosed	32%	
27	F/38	CML-CP	Newly diagnosed	25%	
28	M/57	CML-CP	Newly diagnosed	57%	
29	F/27	CML-CP	Newly diagnosed	115%	
30	F/55	CML-CP	Newly diagnosed	58%	
31	M/18	CML-CP	Newly diagnosed	70%	
32	M/40	CML-CP	Newly diagnosed	65%	
33	M/70	CML-CP	Newly diagnosed	50%	
34	F/72	CML-CP	Newly diagnosed	170%	
35	F/72	CML-CP	Newly diagnosed	81%	
36	M/49	CML-CP	Newly diagnosed	43%	
37	F/35	CML-CP	Newly diagnosed	89%	
38	M/37	CML-CP	Newly diagnosed	90%	
39	F/22	CML-CP	Newly diagnosed	87%	
40	F/27	CML-CP	Newly diagnosed	67%	
41	M/56	CML-CP	Newly diagnosed	81%	
42	M/61	CML-CP	Newly diagnosed	60%	
43	F/50	CML-CP	Newly diagnosed	56%	
44	F/51	CML-CP	Newly diagnosed	82%	
45	M/51	CML-CP	Newly diagnosed	73%	
46	M/64	CML-CP	Newly diagnosed	66%	
